# Approximate nonlinear filtering with a recurrent neural network

**DOI:** 10.1186/1471-2202-16-S1-P196

**Published:** 2015-12-18

**Authors:** Anna Kutschireiter, Simone C Surace, Henning Sprekeler, Jean-Pascal Pfister

**Affiliations:** 1 Institute of Neuroinformatics, University of Zurich and ETH Zurich, 8057 Zurich, Switzerland; 2 Department of Physiology, University of Bern, 3012 Bern, Switzerland; 3 Bernstein Center for Computational Neuroscience, Technical University Berlin, 10587 Berlin, Germany

## 

One of the most fascinating properties of the brain is its ability to continuously extract relevant features in a changing environment. Realizing that sensory inputs are not perfectly reliable, this task becomes even more challenging. This problem can be formalized as a filtering problem where the aim is to infer the state of a dynamically changing hidden variable given some noisy observation. A well-known solution to this problem is the Kalman filter for linear hidden dynamics or the extended Kalman filter for nonlinear dynamics. On the other hand, particle filters offer a sampling-based approach to approximate the posterior distribution. However, it remains unclear how these filtering algorithms may be implemented in neural tissue. Here, we propose a neuronal dynamics which approximates non-linear filtering.

**Figure 1:**
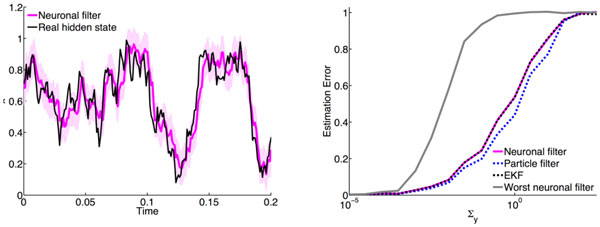
****Left: **A sample trajectory of the real hidden state and its filtered estimate, showing the ability of the neural filter to infer the hidden variable**. **Right: **For a nonlinear hidden dynamics, the neuronal filter we propose achieves an estimation error which is comparable to that of a particle filter or an extended Kalman filter (EKF). The worst neuronal filter corresponds to our filter with a suboptimal parameter choice.

Starting from the formal mathematical solution to the non-linear filter problem, the Kushner equation [1], and assuming linear and noisy observations we derive a stochastic rate-based network whose activity samples the posterior dynamics. We found that taking samples following these stochastic posterior dynamics is able to solve the inference task with a performance comparable to that of standard particle filtering or (extended) Kalman filtering. Indeed, for a linear hidden dynamics we exactly retrieve the Kalman filter equations from our neural filter. In Figure 1 we show the error of the filtered estimate as a function of the observation noise for two different parameter choices in our filter equations.

Thus, the neuronal filter we propose provides an efficient way to infer the state of temporally changing hidden variables. In addition, due to the locality of the underlying mathematical model, the filter is made biologically plausible from a neural-sampling perspective, hence providing a possible framework for the neural sampling hypothesis [2].
